# From meta-analysis to Mendelian randomization: Unidirectional perspectives on the association of glaucoma with depression and anxiety

**DOI:** 10.1371/journal.pone.0310985

**Published:** 2024-11-19

**Authors:** Jie Deng, YuHui Qin

**Affiliations:** 1 First Clinical College of Traditional Chinese Medicine, Hunan University of Chinese Medicine, Changsha, Hunan, China; 2 Graduate School, Hunan University of Chinese Medicine, Changsha, Hunan, China; Brigham and Women’s Hospital, UNITED STATES OF AMERICA

## Abstract

**Background:**

Glaucoma, a primary cause of blindness worldwide, has its association with depression and anxiety noted, yet the understanding of such association is still rudimentary. This study aims to provide the unidirectional perspectives on the association of glaucoma with depression and anxiety, informing public health strategies.

**Methods:**

The Meta-analysis screened observational studies from Medline, Embase, and Web of Science, using the modified Newcastle-Ottawa Scale for quality assessment. It employed R’s ’meta’ package to assess the association between glaucoma and depression or anxiety prevalence. The Mendelian Randomization (MR) analysis was conducted using R’s ’TwoSampleMR’ package, based on data from the IEU database data to explore the impact of glaucoma on depression and anxiety.

**Results:**

This Meta-analysis included 23 out of 379 studies involving 11,845 glaucoma patients. The combined prevalence of depression among glaucoma patients, derived from 18 cross-sectional studies, stood at 19.42%. Five case-control studies indicated that glaucoma patients had a 6.17-fold higher risk of depression compared to controls. Derived from 16 cross-sectional studies, the consolidated prevalence for anxiety was 19.07%. According to five case-control studies, glaucoma patients exhibited a 4.45-fold increased risk of anxiety compared to controls. MR analysis failed to uncover a causal effect of glaucoma on depression and anxiety.

**Conclusion:**

This study suggests that glaucoma patients may experience higher prevalence of depression and anxiety than the general population, with no clear genetic links found. It suggests that environmental factors and non-genetic biological pathways, among others, may play significant roles in their association, though the role of genetic factors cannot be ruled out. These findings highlight the necessity of a comprehensive approach to study the complex factors influencing the association of glaucoma with depression or anxiety and underscore the importance of integrating mental health considerations into glaucoma management to improve medication adherence and disease progression.

## Introduction

Glaucoma, characterized by impaired aqueous humor drainage, elevated intraocular pressure, and optic nerve damage, frequently leads to blindness [1]. Currently, approximately 76 million individuals globally are affected by glaucoma, a number expected to rise to 112 million by 2040 [2]. Glaucoma, presenting in various forms, can lead to severe visual impairment, and may also include systemic symptoms [3]. Patients with glaucoma also encounter psychological challenges, such as anxiety and depression, which affect their mental and physical health. Consequently, glaucoma is a major public health concern worldwide.

It is well-known that psychological states significantly influence the development, progression, and resolution of diseases. Numerous studies have observed glaucoma association with depression and anxiety, with most studies suggesting that glaucoma may lead to depression and anxiety [4–6]. However, current insights remain cursory, primarily framing the disease as a stressor that precipitates depression and anxiety. These viewpoints are predominantly subjective, without the support of rigorous experimental and scientific proof. Moreover, they overlook other potential factors. Despite increasing interest in the association of glaucoma with depression and anxiety, a comprehensive and deep understanding of its nature and mechanisms remains absent. Furthermore, the prevalence of depression and anxiety among glaucoma patients significantly differ across studies. Given that this link might involve subtypes of the disease, severity, genetics, environmental influences, and beyond, pinpointing the primary factors holds substantial practical and theoretical importance.

Although numerous factors contribute to the development of glaucoma, depression, and anxiety, genetic and environmental elements are considered pivotal [7–9]. Genetics significantly influence susceptibility to glaucoma, depression, and anxiety. Additionally, A diverse array of environmental factors, such as social and family environments, economic pressures, and personal lifestyles, also profoundly impact the incidence of these conditions. The association of glaucoma with depression and anxiety may be attributed to genetic factors, environmental factors, their interaction, or shared underlying mechanisms. Investigating the association of glaucoma with depression and anxiety, along with related factors, can enhance psychological support, facilitate early diagnosis, inform public health policies, and improve education efforts.

This study utilizes Meta-analysis to explore the associations between glaucoma and depression or anxiety prevalence, and MR analysis to explore potential causal effects, preliminary exploring the related factors. Meta-analysis aggregates multiple independent studies to provide overarching conclusions on specific research questions. By enlarging the sample size, it enhances statistical power and result credibility while reducing random errors, and provides accurate and reliable estimates, critical for addressing inconsistencies in research findings. Meta-analysis is versatile, including observational studies and randomized controlled trials (RCTs), etc. [10]. MR, grounded in genome-wide association studies (GWAS), infers potential causal relationships between exposures and outcomes. It uses single nucleotide polymorphisms (SNPs) as instrumental variables (IVs), leveraging the random allocation of alleles to eliminate confounders and avoid reverse causality. The inherent stability and environmental independence of genetic information enhance the reliability of MR, significantly reducing potential biases [11].

Given the challenges and high costs of conducting RCTs in this field, the combined use of Meta-analysis and MR provides significant advantages. This approach not only enriches data interpretation but also provide the unidirectional perspectives on associations of glaucoma with depression and anxiety, thereby deepening our understanding of the involved mechanisms and filling knowledge gaps. This research contributes new insights that inform public health policies on glaucoma and guide future interventions.

## Methods

### Meta-analysis

#### Data sources and retrieval

We conducted a comprehensive literature search across Medline, Embase, and Web of Science databases without restrictions on language or publication date, supplementing it by reviewing references of relevant articles, on December 20, 2023. We utilized PubMed for Medline searches, using a combination of keywords, MeSH terms, and text words. The search strategy was: ("Glaucoma"[Mesh] OR "Open-Angle Glaucoma"[Mesh] OR "Angle-Closure Glaucoma"[Mesh] OR glaucoma OR "open angle glaucoma" OR "angle closure glaucoma") AND ("Depression"[Mesh] OR depression OR "Depressive Disorder"[Mesh]) AND ("Anxiety"[Mesh] OR anxiety OR "Anxiety Disorders"[Mesh]) AND ("Observational Study"[Mesh] OR cohort OR "cross-sectional" OR "case-control"). The Embase search strategy was: (’glaucoma’/exp OR ’open angle glaucoma’/exp OR ’angle closure glaucoma’/exp OR glaucoma OR ’open angle glaucoma’ OR ’angle closure glaucoma’) AND (’depression’/exp OR depression OR ’depressive disorder’/exp) AND (’anxiety’/exp OR anxiety OR ’anxiety disorder’/exp) AND (’observational study’/exp OR ’cohort analysis’/exp OR ’cross sectional study’/exp OR ’case control study’/exp). The search formula in Web of Science was: TS = ("Glaucoma" OR "Open-Angle Glaucoma" OR "Angle-Closure Glaucoma") AND TS = ("Depression" OR "Depressive Disorder") AND TS = ("Anxiety" OR "Anxiety Disorders")." S1 Fig displays the complete PRISMA flow diagram.

#### Study selection

Following PRISMA guidelines, two authors independently reviewed titles and abstracts for eligibility, then conducted full-text screening for final selection, resolving discrepancies through discussion. Inclusion criteria included: (1) observational study designs; (2) provision of sample sizes, prevalence rates, or calculation from raw data; (3) undergoing some form of quality assessment; (4) clear definition of glaucoma, depression and anxiety. Exclusion criteria included: (1) conference proceedings, case reports, reviews, meta-analyses; (2) studies with incomplete data, making it impossible to calculate necessary metrics; (3) subjects with other diseases significantly affecting mental health; (4) studies of poor quality.

#### Data extraction and quality assessment

Two authors independently extracted data including study title, type, publication year, demographics (gender, age, population), diagnostic criteria, sample size, and prevalence. Study quality was assessed using the modified Newcastle-Ottawa Scale (NOS) [12], as outlined in S1 Table. The NOS scale evaluates quality based on criteria like sample selection, study design, outcome assessment, confounder assessment, data analysis, and study results. Scoring criteria were: 0 points (not met), 1 point (partially met), and 2 points (fully met). Based on total scores, Studies were classified into low (11–14 points), medium (6–10 points), or high (0–5 points) bias risk categories.

#### Statistical analysis

We conducted the analysis using R’s ’meta’ package (version 4.1.1). Random-intercept logistic regression models assessed the overall prevalence of depression or anxiety in cross-sectional studies, while the inverse variance method estimated effect sizes in case-control studies. Heterogeneity was assessed using Cochran’s Q test; a P-value <0.05 indicated the presence of heterogeneity, followed by the I^2^ test for its extent, and restricted maximum likelihood for variance estimation. Significant heterogeneity (I^2^ ≥ 50%) necessitated the use of random-effects models; otherwise, fixed-effects models were employed. Publication bias was assessed with Funnel plots and Egger’s Test. Leave-One-Out analysis sequentially excluded each study to ensure result stability and consistency. Meta-analysis results were displayed in forest plots, along with Odds Ratios (OR) and 95% confidence intervals (CI).

### MR analysis

#### Study design

**Fig 1:**
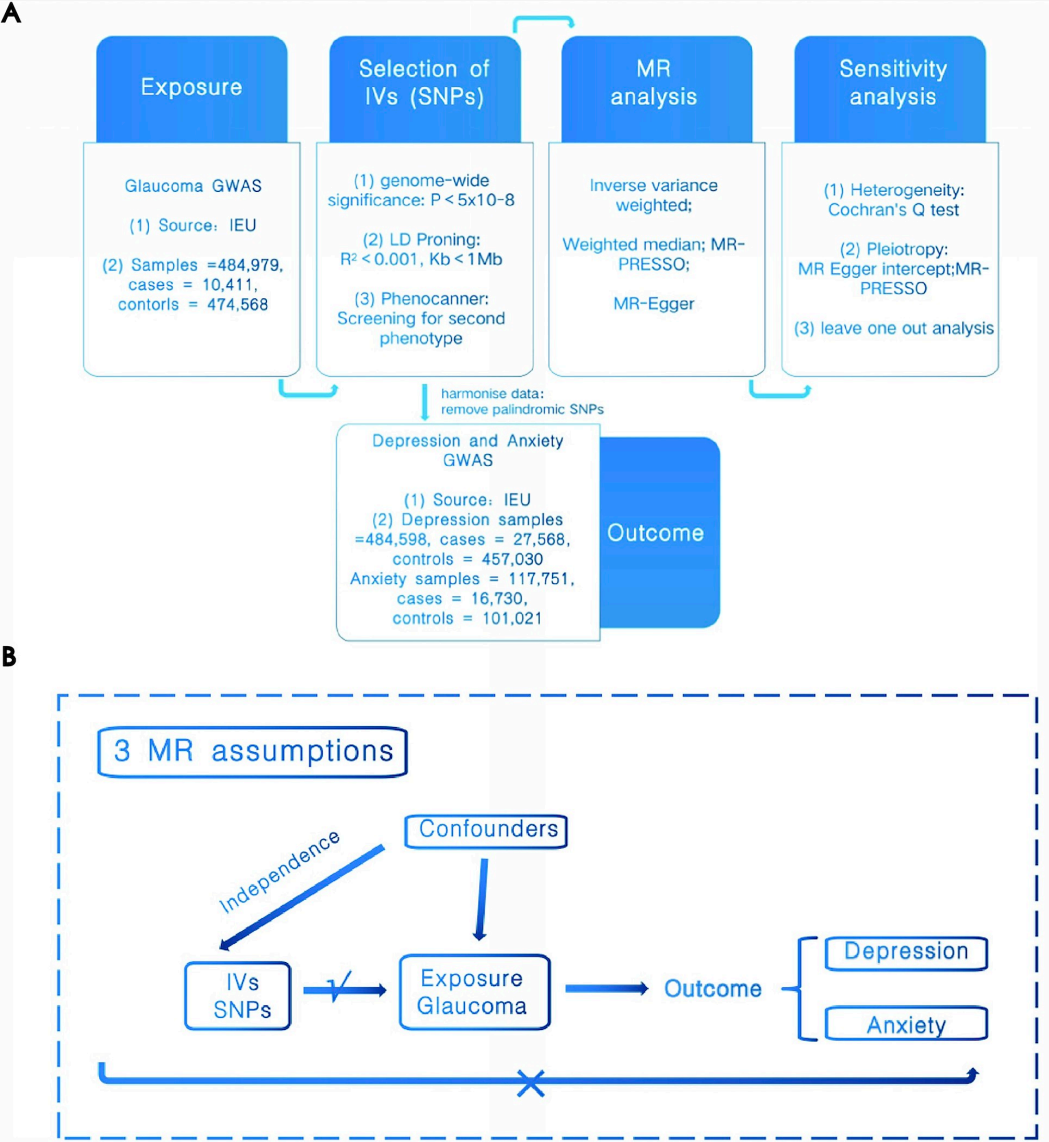
(A) Overview of MR analyses. (B) Major assumptions of MR.

We performed a two-sample MR analysis to investigate the effects of glaucoma on depression and anxiety using public GWAS database, as depicted in Fig 1A. Selected genetic variants closely associated with glaucoma served as IVs for exploring its causal effects on depression and anxiety. The MR analysis followed three critical assumptions [13] detailed in Fig 1B: (1) SNPs must be strongly correlated with glaucoma; (2) These SNPs should be independent of any confounders; (3) The influence on depression or anxiety must occur solely through these SNPs, with no direct links or alternative pathways. The utilized GWAS data were derived from the Integrative Epidemiology Unit (IEU) database, did not require Ethics board approval as per its guidelines.

#### GWAS data sources

All GWAS data were sourced from the IEU database. Glaucoma GWAS data originated from a large-scale GWAS analysis of European ancestry by Sakaue S et al., published in 2021 (ID: ebi-a-GCST90018852), with a sample size of 484,979, including 10,411 cases and 474,568 controls [14]. Depression data were from a large-scale GWAS study by Handan MD, Daniel KF et al., in 2021 (ID: ebi-a-GCST90038650), involving 484,598 participants with 27,568 cases and 457,030 controls [15]. Anxiety data were derived from a major GWAS analysis using UK Biobank data, conducted by Neale’s lab in 2018 (ID: ukb-d-20544_15), comprising 117,751 individuals, with 16,730 cases and 101,021 controls (https://gwas.mrcieu.ac.uk/).

#### IV selection criteria

To fulfill MR assumptions, firstly, SNPs linked to the exposure were selected based on a stringent significance threshold (P < 5×10^-8). Linkage disequilibrium analysis was then conducted based on independence criteria (LD value r^2^ < 0.001, Kb < 1Mb) to exclude ineligible SNPs. Secondly, each exposed SNP should independently influence the outcome; hence, secondary phenotypes of SNPs were filtered using the Phenoscanner website (http://www.phenoscanner.medschl.cam.ac.uk/), manually removing SNPs related to the outcomes (P < 5×10^-8). Harmoniza analysis ensured alignment between allelic directions of exposure and outcome SNPs, excluding palindromic SNPs with indeterminable directions and incompatible SNPs. Finally, the strength of IVs was quantified using the F-statistic, with F > 10 indicating sufficient strength. The formula for calculation is R^2^ = 2*(1-EAF)EAF*(β)^2^, F = (R^2^/1-R^2^)(n-k-1/k), where R^2^ is the total proportion of variance in phenotype explained by all genetic variants in exposure, EAF is the effect allele frequency, β is the beta value of SNP, k is the number of SNPs, and n is the sample size.

#### Statistical analysis

We conducted MR analysis to explore glaucoma’s causal impact on depression and anxiety using the ’TwoSampleMR’ package in R software (version 4.1.1). The Inverse Variance Weighted (IVW) method with a random-effects model were employed to ensure the accuracy and sample size of each SNP were adequately considered. Results were displayed as OR with 95% CI. Beyond the IVW method, supplementary MR analyses included Weighted Median (WM), MR-PRESSO, and MR-Egger regression. An association was considered significant if the IVW method’s P-value was below 0.05. The reliability of results was further confirmed when both WM and MR-Egger P-values were under 0.05. However, the absence of significance in these additional methods did not negate the results’ reliability, as they might be influenced by certain limitations or assumptions. To evaluate MR results’ robustness, we performed sensitivity analyses including Cochran’s Q test, MR-Egger intercept analysis, and Leave-One-Out analysis. Initially, Cochran’s Q test was used to check for heterogeneity among IVs. A P-value > 0.05 indicated no significant heterogeneity among them. Even in the presence of heterogeneity, the IVW method could still provide accurate estimates due to our use of a random-effects model. MR-Egger intercept analysis assessed the presence of horizontal pleiotropy, with P < 0.05 indicating its presence. To further ensure the accuracy of causal relationships, MR-PRESSO was utilized to detect biased IVs, thereby mitigating heterogeneity. Additionally, Leave-One-Out analysis was used as a complementary sensitivity analysis, sequentially excluding each IV and observing the consistency and stability of the results.

## Results

### Meta-analysis

#### Study characteristics

The literature search identified 382 studies. Following the inclusion and exclusion criteria, 355 articles were excluded after reviewing titles and abstracts, and 4 more were eliminated after full-text assessments. A comprehensive list of all studies identified in the literature search, including the reasons for exclusions, is provided in S2 Table. The statistical analysis incorporated 23 studies [16–38], comprising 18 cross-sectional and 5 case-control studies. All studies were incorporated into a meta-analysis on the association between glaucoma and depression prevalence, while 21 of these studies also examined its association with anxiety prevalence. Twelve studies employed the Hospital Anxiety and Depression Scale (HADS), three utilized the Patient Health Questionnaire-9 (PHQ-9) and Generalized Anxiety Disorder-7 (GAD-7). One study each employed the Self-Rating Anxiety Scale (SAS) and Self-Rating Depression Scale (SDS), the Beck Depression Inventory-II (BDI-II) combined with State-Trait Anxiety Inventory (STAI), and the Beck Anxiety Inventory (BAI) with the BDI-II, while another used the Geriatric Depression Scale-15 (GDS-15). Additionally, one study used the BDI-II. Two studies were based on Electronic Health Records (EHR), including billing codes, medical history, and problem lists. One study employed the revised Clinical Interview Schedule (CIS-R). Baseline characteristics of the included trials are detailed in Table 1. Some studies in the Table 1 have missing data, but these are not essential for this research. Thus, the missing data are unlikely to impact the overall conclusions of the meta-analysis.

**Table 1:** Basic characteristics of studies.

Study ID	Study Type	Publication Year	Age Range	Gender Distribution	Sample Size	Assessment Tool	Depression Cases	Anxiety Cases	Data Extractor	Data Extraction Date	Inclusion Eligibility
1[16]	Cross-sectional Study	2021	70.14 ± 15.8	Male 74, Female 55	129	HADS	7	10	Jie Deng	2023-12-21	YES
2 [17]	Case-control Study	2022	CG: 57.00 ± 10.15, CtrlG: 62.47 ± 9.29	CG: Male 81, Female 67, CtrlG: Male 75, Female 75	CG: 148, CtrlG: 150	PHQ-9, GAD-7	CG: 53, CtrlG: 25	CG: 37, CtrlG: 18	Jie Deng	2023-12-21	YES
3 [18]	Cross-sectional Study	2020	59.7 ± 13.3	Male 74,Female 108	182	HADS	76	80	Jie Deng	2023-12-21	YES
4 [19]	Cross-sectional Study	2021	67.1 ± 12.0	Male 61, Female 39	100	PHQ-9, GAD-7	30	64	Jie Deng	2023-12-21	YES
5 [20]	Cross-sectional Study	2013	55.40 ± 15.26	Male 263	506	HADS	81	116	Jie Deng	2023-12-21	YES
6 [21]	Case-control Study	2015	PACG: 58.16 ± 14.42, POAG: 52.86 ± 12.64	CG: Male 52, Female 48, CtrlG: Male 25, Female 25	CG: 100, CtrlG: 50	SAS, SDS	CG: 48, CtrlG: 5	CG: 55, CtrlG: 8	Jie Deng	2023-12-23	YES
7 [22]	Cross-sectional Study	2018	35–74	Male 25, Female 145	293	PHQ-9, GAD-7	19	16	Jie Deng	2023-12-23	YES
8 [23]	Case-control Study	2010	CG: 64.23 ± 13.2, CtrlG: 61.43 ± 9.55	CG: Male 53, Female 68, CtrlG: Male 23, Female 41	CG: 121, CtrlG: 64	HADS	CG: 69, CtrlG: 6	CG: 17, CtrlG: 2	Jie Deng	2023-12-23	YES
9 [24]	Case-control Study	2008	CG: 66.9 ± 11.9, CtrlG: 67.4 ± 12.1	CG: Male 88, Female 142, CtrlG: Male 88, Female 142	CG: 230, CtrlG: 230	HADS	CG: 25, CtrlG: 12	CG: 30, CtrlG: 16	Jie Deng	2023-12-23	YES
10 [25]	Cross-sectional Study	2014	56.5 ± 17.2	Male 266,Female 234	500	HADS	130	56	Jie Deng	2023-12-23	YES
11 [26]	Cross-sectional Study	2013	70.8	Male 43, Female 43	86	BDI-II, STAI	31	18	Jie Deng	2023-12-24	YES
12 [27]	Cross-sectional Study	2021	53.23 ± 13.03	Male 107, Female 144	251	BAI, BDI-II	40	44	Jie Deng	2023-12-24	YES
13 [28]	Cross-sectional Study	2018	72.9 ± 9.8	Male 32, Female 57	89	GDS-15	16	Not Provided	Jie Deng	2023-12-24	YES
14 [29]	Cross-sectional Study	2022	67.5 ± 13.3	Male 100, Female 76	176	HADS	12	16	Jie Deng	2023-12-24	YES
15 [30]	Cross-sectional Study	2021	67.6±13.8	Not Provided	111	BDI-II	56	Not Provided	Jie Deng	2023-12-24	YES
16 [31]	Case-control Study	2020	CG: 58.14 ± 13.88, CtrlG: 57.19±13.76	CG: Male 66, Female 114, CtrlG: Male 66, Female 114	CG: 180, CtrlG: 180	HADS	CG: 39, CtrlG: 2	CG: 59, CtrlG: 6	Jie Deng	2023-12-24	YES
17 [32]	Cross-sectional Study	2018	57.20 ±13.94	Male 140, Female 123	263	HADS	73	78	Jie Deng	2023-12-24	YES
18 [33]	Cross-sectional Study	2022	57.40±15.99	Male 232, Female 214	446	HADS	115	54	Jie Deng	2023-12-24	YES
19 [34]	Cross-sectional Study	2021	59.75±8.43	Male 29, Female 53	182	HADS	25	33	Jie Deng	2023-12-25	YES
20 [35]	Cross-sectional Study	2021	60.0	Male 383, Female 528	911	EHR	300	291	Jie Deng	2023-12-25	YES
21 [36]	Cross-sectional Study	2020	56.6 ± 15.7	Male 42, Female 22	64	HADS	10	18	Jie Deng	2023-12-25	YES
22[37]	Cross-sectional Study	2021	58 ± 9.0	Male 123, Female 180	303	CIS-R	13	47	Jie Deng	2023-12-25	YES
23[38]	Cross-sectional Study	2020	Not Provided	Not Provided	5800	EHR	1876	651	Jie Deng	2023-12-25	YES

CG: Case Group; CtrlG: Control Group.

#### Quality assessment

S3 Table presents the quality assessment for all studies using the modified NOS. Eleven studies were classified as low risk of bias, and thirteen as moderate risk.

#### Comprehensive interpretation

Twenty-three studies, covering 11,845 glaucoma patients, assessed the association between glaucoma and depression prevalence, with 3,194 patients reporting depression, leading to a combined prevalence of 26.97%. Of these, twenty-one studies further analyzed the association between glaucoma and anxiety prevalence, involving 11,645 patients from the original cohort, 1,840 of whom reported anxiety, resulting in a prevalence of 15.80%.

#### Association between glaucoma and depression prevalence: Cross-sectional study results

**Fig 2:**
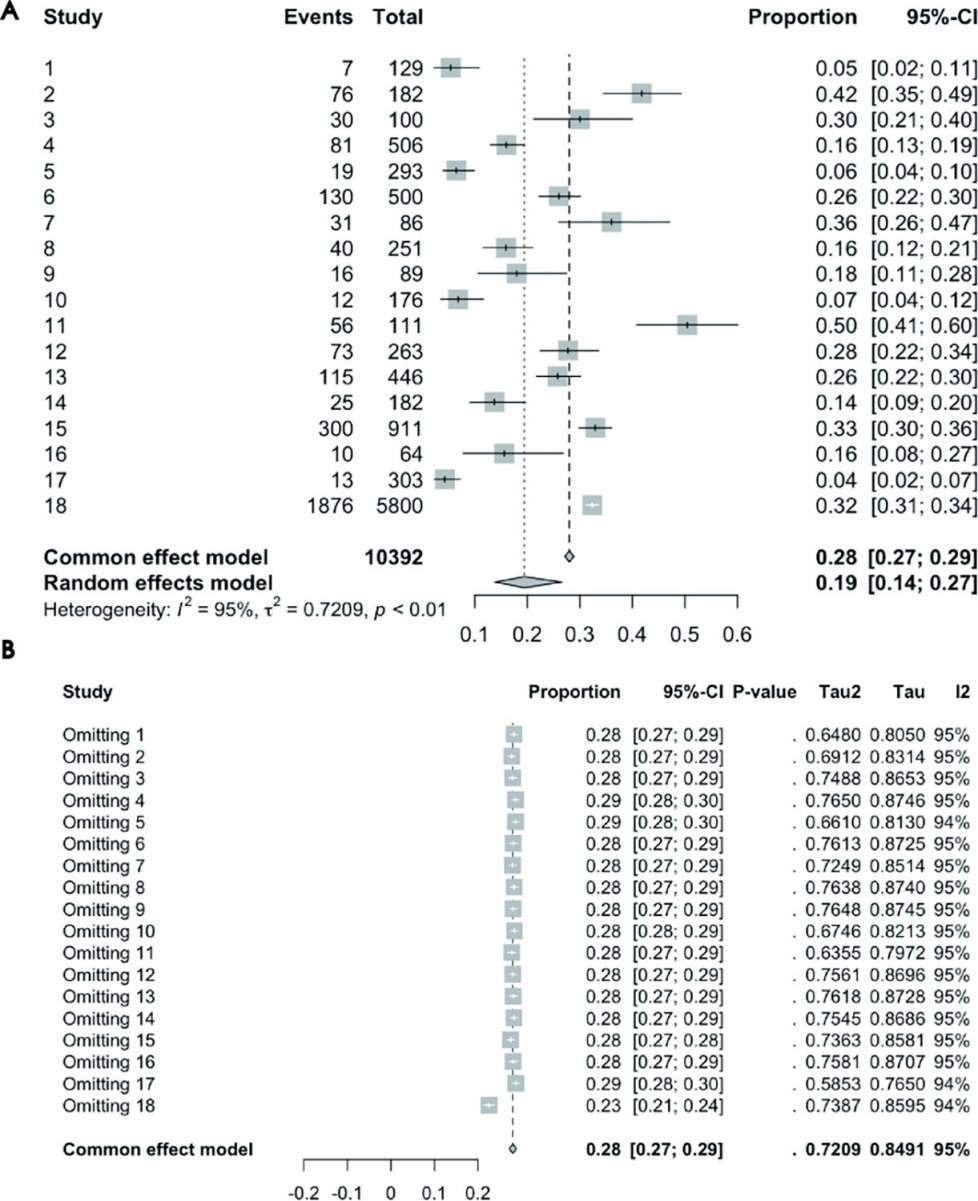
(A) Forest Plot of Cross-sectional Studies on the Association Between Glaucoma and Depression Prevalence. (B) Leave-One-Out Analysis Forest Plot for Cross-sectional Studies on the Association Between Glaucoma and Depression Prevalence.

Data from 18 cross-sectional studies were collectively analyzed. Cochran’s Q test initially revealed significant heterogeneity (P < 0.0001). The restricted maximum likelihood method further confirmed significant study variance (τ^2^ = 0.7209). An I^2^ value of 95.1% from the I2 test, indicative of considerable heterogeneity, necessitated using a random-effects model. Under this model, utilizing random-intercept logistic regression, yielded a combined depression prevalence of 19.42% (95% CI = 13.86% - 26.54%). Fig 2A displays the forest plot of the results.

*Sensitivity Analysis Results*. The Leave-One-Out analysis forest plot (Fig 2B) demonstrates that while individual studies influenced the overall estimate, none significantly altered the meta-analysis results. Regarding heterogeneity, the I^2^ values remained high even after omitting any study, signifying significant heterogeneity among the studies, most over 70%.

*Publication bias results*. Fig 6A presents the funnel plot for cross-sectional studies assessing the association between glaucoma and depression prevalence. Egger’s Test revealed significant publication bias (t = -3.57, df = 18, p-value = 0.0022). These results suggest that smaller effect sizes or non-significant studies might have remained unpublished, potentially affecting the accuracy of the overall results.

#### Association between glaucoma and depression prevalence: Case-control study results

**Fig 3:**
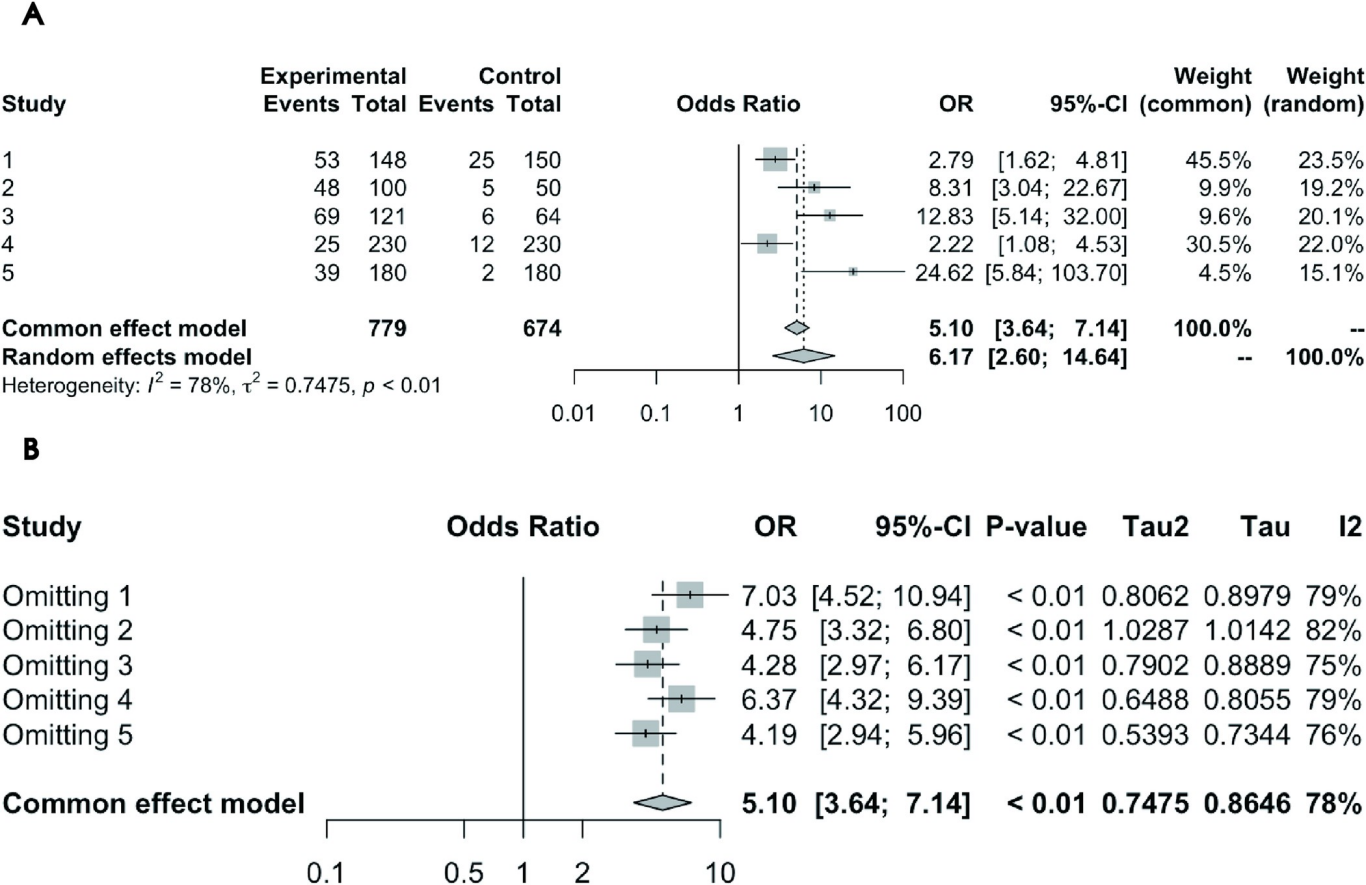
(A) Forest Plot of Case-Control Studies on the Association Between Glaucoma and Depression Prevalence. (B) Leave-One-Out Analysis Forest Plot for Case-Control Studies on the Association Between Glaucoma and Depression Prevalence.

Data from 5 case-control studies were collectively analyzed. Cochran’s Q test initially revealed significant heterogeneity (P = 0.0010). Further calculations using the restricted maximum likelihood method confirmed significant studies variance (τ^2^ = 0.7475). The I^2^ test revealed substantial heterogeneity (I^2^ value = 78.4%), necessitating a random-effects model. Under this model, employing the inverse variance method revealed that glaucoma patients had a 6.17 times higher risk of depression compared to controls (OR = 6.1737, 95% CI = 2.6035–14.6399). Fig 3A displays the forest plot of the results.

*Sensitivity Analysis Results*. The Leave-One-Out analysis forest plot (Fig 3B) shows that while individual studies influenced the overall estimate, none significantly altered the meta-analysis results. Regarding heterogeneity, the I^2^ values remained high (the lowest being 75.3%) even after omitting any study, signifying significant heterogeneity among the studies.

*Publication Bias Results*. Egger’s Test was not applicable due to the limited number of studies. Fig 6B displays the funnel plot from the 5 case-control studies on the association between glaucoma and depression prevalence. The asymmetry in the funnel plot suggests that smaller effect sizes or non-significant studies might have remained unpublished, potentially affecting the accuracy of the overall results.

#### Association between glaucoma and anxiety prevalence: Cross-sectional study results

**Fig 4:**
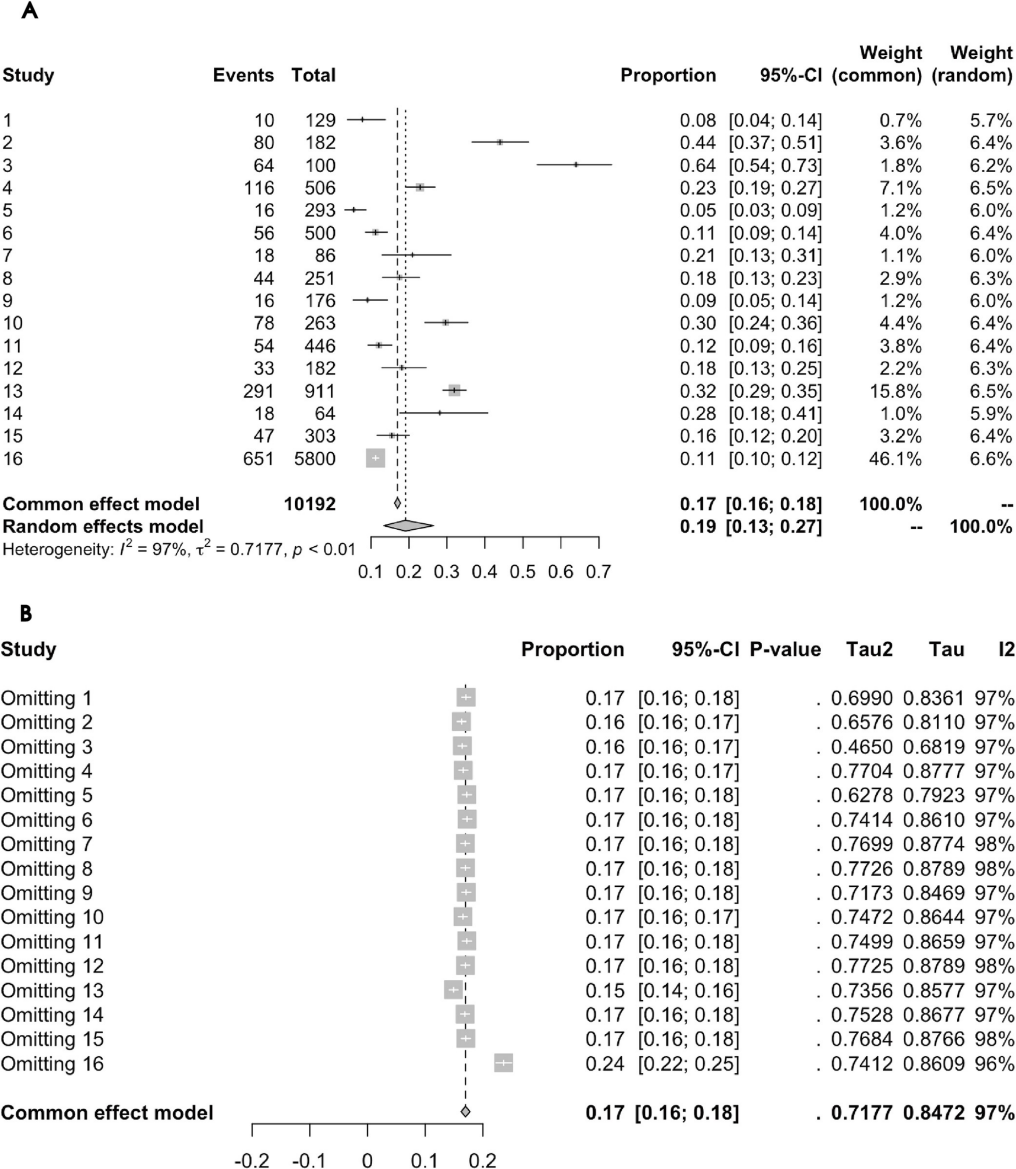
(A) Forest Plot of Cross-sectional Studies on the Association Between Glaucoma on Anxiety Prevalence. (B) Leave-One-Out Analysis Forest Plot for Cross-sectional Studies on the Association Between Glaucoma and Anxiety Prevalence.

Data from 16 cross-sectional studies, underwent combined analysis. Cochran’s Q test initially showed significant heterogeneity (P < 0.0001). Further calculation using the restricted maximum likelihood method indicated significant heterogeneity among studies (τ^2^ = 0.7177). The I^2^ test showed an I^2^ value of 97.3%, indicating significant heterogeneity, which led to the adoption of a random-effects model. Under this model, using random-intercept logistic regression, the combined anxiety prevalence in glaucoma patients was found to be 19.07% (95% CI = 13.34% - 26.51%). Fig 4A displays the forest plot of the results.

*Sensitivity Analysis Results*. The Leave-One-Out analysis forest plot (Fig 4B) demonstrates that although individual studies influenced the overall estimate, none had a decisive impact on the overall results. Regarding heterogeneity, even with the omission of any study, the I^2^ values remained extremely high, with the lowest being 96%. This indicates significant differences among the studies.

*Publication Bias Results*. Fig 6C shows the funnel plot for the 16 Cross-sectional studies on the Impact of glaucoma on anxiety. The results of Egger’s Test indicated no significant publication bias statistically (t = 1.02, df = 15, p-value = 0.3217).

#### Association between glaucoma and anxiety prevalence: Case-control study results

**Fig 5:**
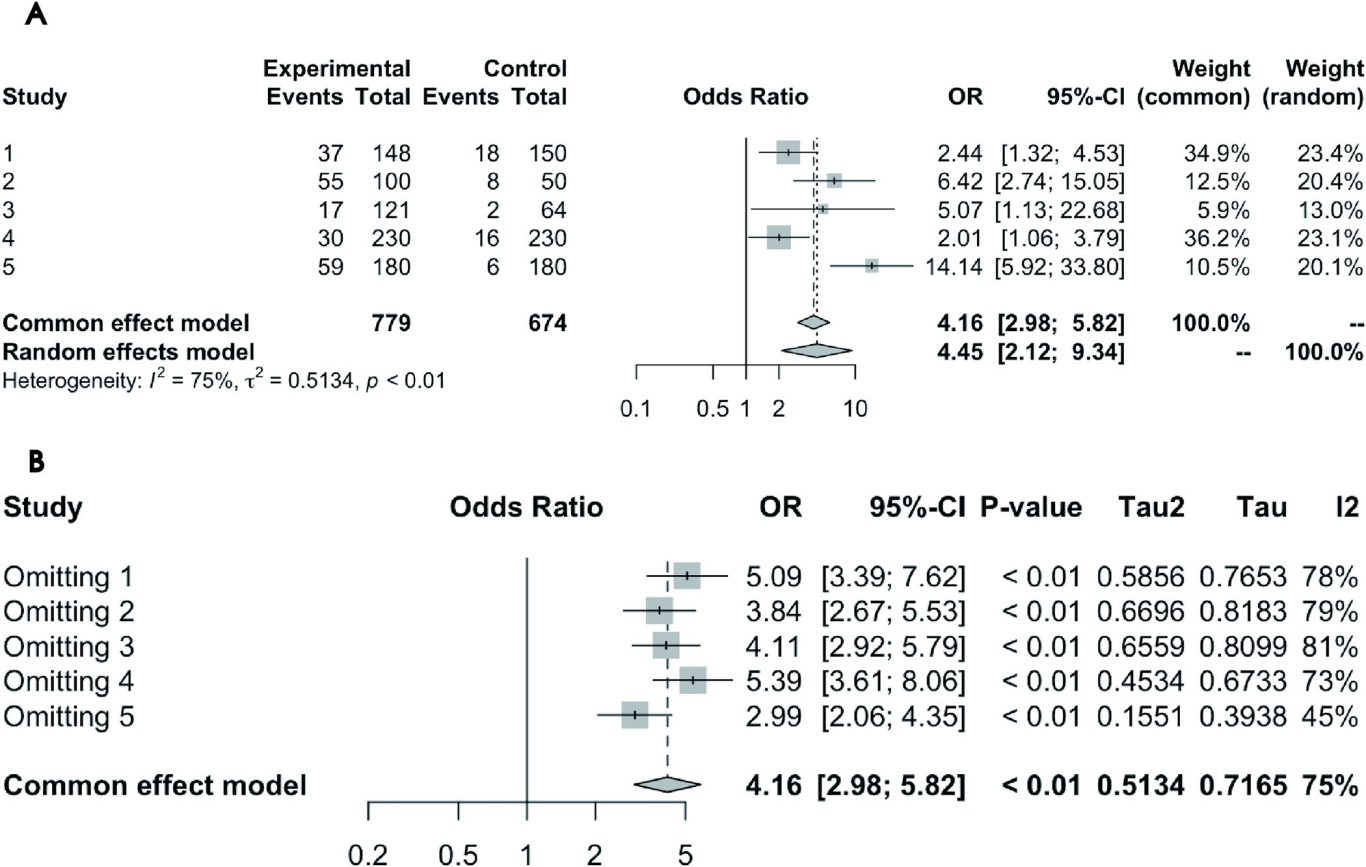
(A) Forest Plot of Case-Control Studies on the Association Between Glaucoma on Anxiety Prevalence. (B) Leave-One-Out Analysis Forest Plot for Case-Control Studies on the Association Between Glaucoma and Anxiety Prevalence.

Data from 5 case-control studies were collectively analyzed. Cochran’s Q test initially showed significant heterogeneity (P = 0.0028). Subsequently, heterogeneity variance (τ^2^ = 0.7165) was calculated using the restricted maximum likelihood method, confirming significant study differences. The I^2^ test revealed substantial heterogeneity with an I^2^ value of 75.2%, leading to the adoption of a random-effects model. Using the inverse variance method under this model, glaucoma patients were found to have a 4.45 times higher risk of anxiety compared to controls (OR = 4.4501, 95% CI = 2.6035–14.6399). Fig 5A displays the forest plot of the results.

*Sensitivity Analysis Results*. The Leave-One-Out analysis forest plot (Fig 5B) indicates that although individual studies influenced the overall estimate, none had a decisive impact on the overall results. Regarding heterogeneity, the I^2^ values remained high even after omitting any study (the lowest being 72.6%), signifying significant differences among the studies.

**Fig 6:**
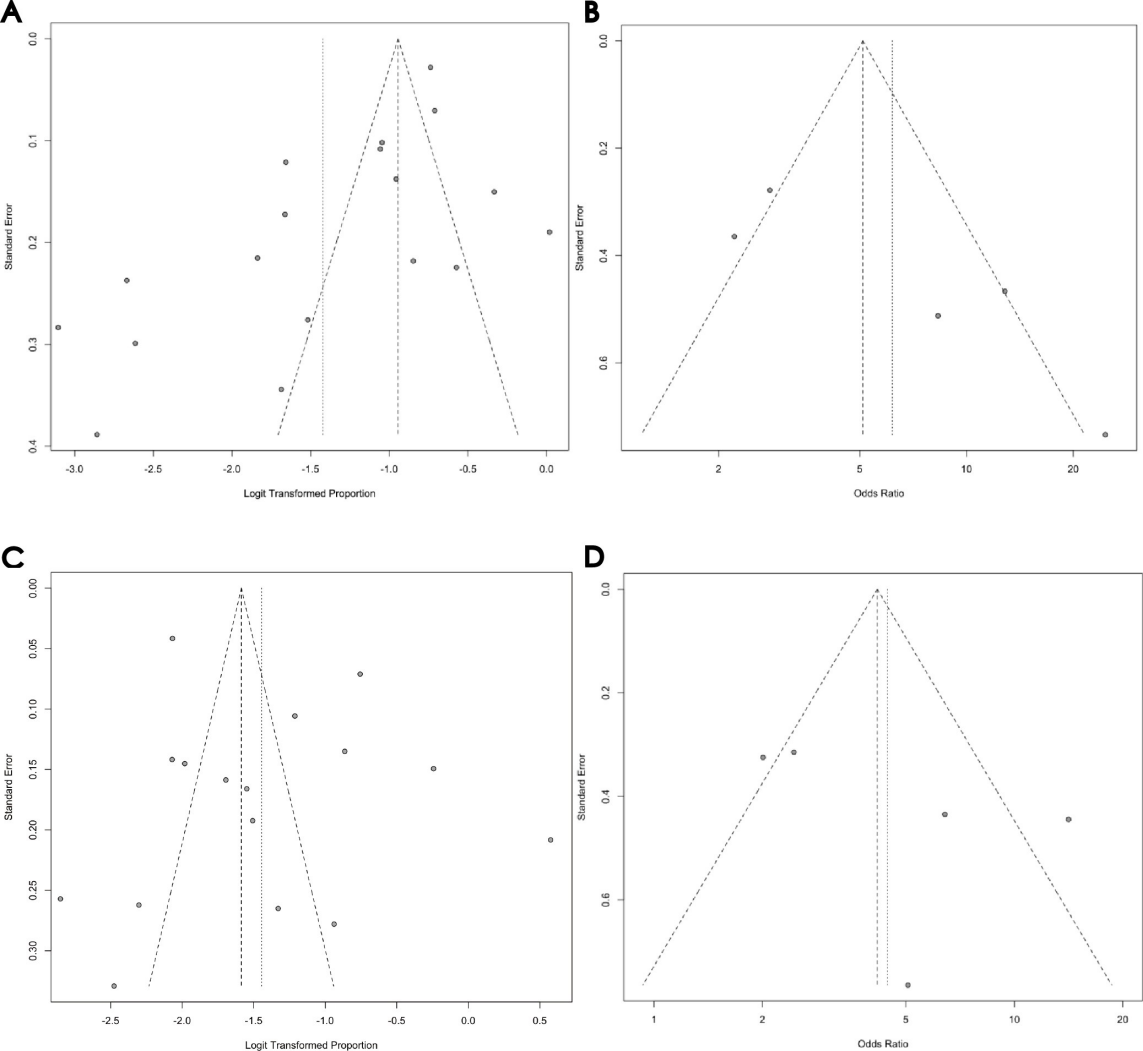
(A) Funnel Plot of Cross-sectional Studies on the Association Between Glaucoma and Depression Prevalence. (B) Funnel Plot of Case-Control Studies on the Association Between Glaucoma and Depression Prevalence. (C) Funnel Plot of Cross-sectional Studies on the Association Between Glaucoma and Anxiety Prevalence. (D) Funnel Plot of Case-Control Studies on the Association Between Glaucoma and Anxiety Prevalence.

*Publication Bias Results*. Egger’s Test was not conducted due to the limited number of studies. Fig 6D shows the funnel plot for the 5 case-control studies on the association between glaucoma and anxiety prevalence. The asymmetry in the funnel plot suggests the possibility of unpublished studies with smaller effect sizes or non-significant results, potentially impacting the accuracy of the overall findings.

### MR analysis

#### Selection of glaucoma-related IVs

After P-value filtering and removing linkage disequilibrium via LD effect, 34 SNPs from the GWAS data on glaucoma were identified as both significantly associated and independent. Harmonization analysis, which included palindromic quality control, led to the exclusion of 2 glaucoma-related SNPs (rs4899012, rs9853115), yielding a final count of 32 SNPs. The average F-statistic for the selected SNPs was 192.348, well above the threshold of 10, indicating sufficient strength of these SNPs as IVs.

#### MR results

**Fig 7:**
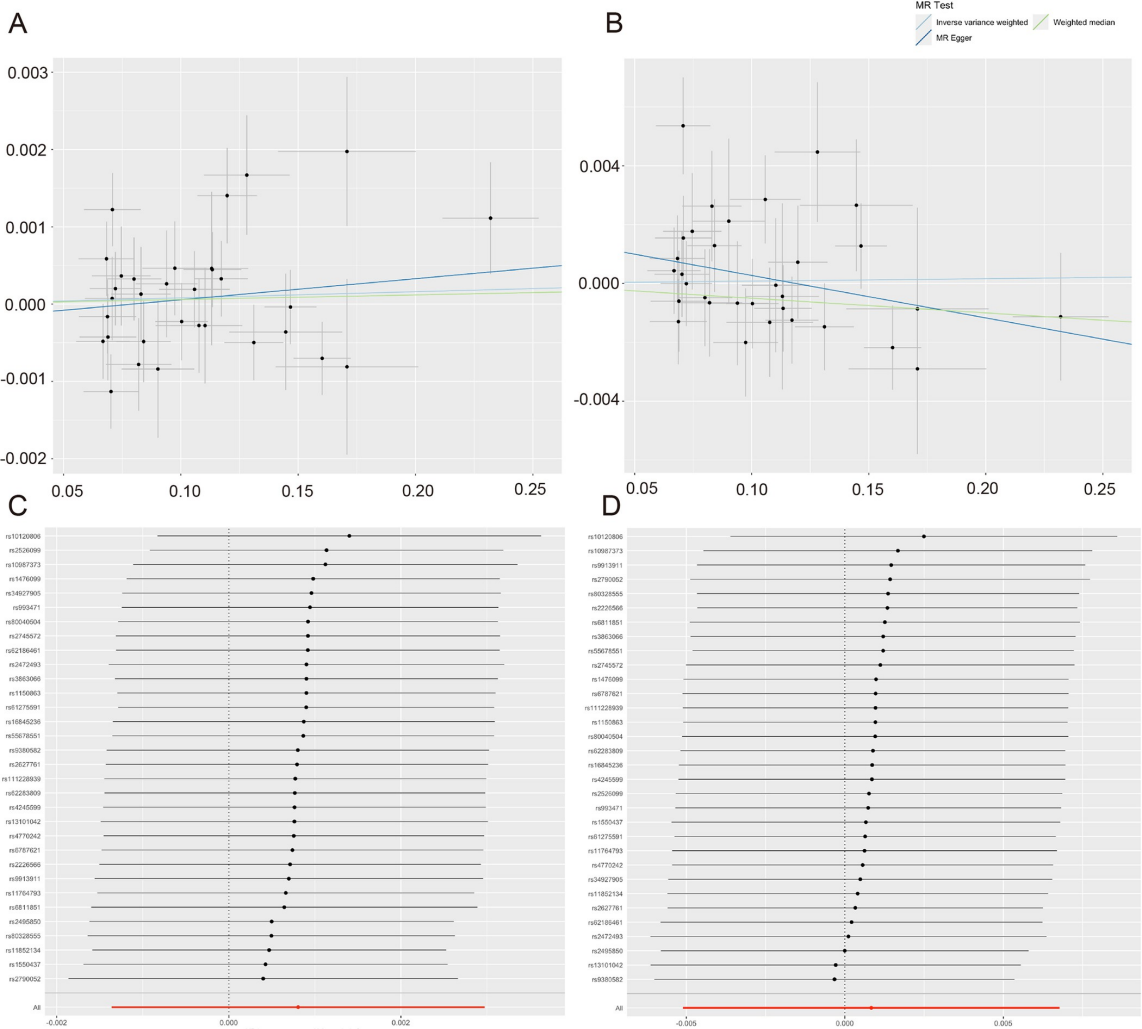
(A) Scatter plot of Glaucoma-related SNPs’ causal effects on Depression. (B) Scatter plot of Glaucoma-related SNPs’ causal effects on Anxiety. (C) Leave-One-Out analysis Forest plot of Glaucoma-related SNPs’ causal effects on Depression. (D) Leave-One-Out analysis Forest Plot of Glaucoma-related SNPs’ causal effects on Anxiety. Light blue line: IVW method estimate. Dark blue line: MR-Egger regression estimate. Grass green line: Weighted Median estimate. Circles represent genetic associations; error bars indicate 95% CIs. Black dots and lines show individual SNP causal effects and CIs. Red dots and lines at bottom indicate overall effects and CIs. X-axis in log scale.

The IVW model revealed no causal effect of glaucoma on depression (OR = 1.000, 95% CI = 0.998–1.002, P = 0.466) or anxiety (OR = 1.000, 95% CI = 0.994–1.006; P = 0.783). Consistent results were also observed with the WM and MR-Egger methods. Table 2 presents the detailed data. Scatter plots (Fig 7A and 7B) illustrate the causal inferences for individual IVs.

**Table 2:** MR Estimates for glaucoma’s effects on depression and anxiety.

Outcomes	Particpants samples	MR estimates, per log-odds ratio increment					
		Inverse variance weighted		Weighted median		MR-Egger	
		OR(95% CI)	P-value	OR(95% CI)	P-value	OR(95% CI)	P-value
Depression	484,598	1.000(0.998–1.002)	0.466	1.000(0.997–1.003)	0.673	1.002(0.995–1.009)	0.435
Anxiety	117,751	1.000(0.994–1.006)	0.783	0.995(0.986–1.003)	0.245	0.985(0.968–1.003)	0.119

#### Sensitivity analysis results

Table 3 displays the analysis of heterogeneity and pleiotropy regarding glaucoma’s effects on depression and anxiety. Under the IVW model, Cochran’s Q test showed no significant heterogeneity in glaucoma’s effects on depression (P = 0.085) and anxiety (P = 0.306). MR-Egger intercept analysis revealed no evidence of horizontal pleiotropy for glaucoma with respect to depression (P = 0.560) and anxiety (P = 0.082). No biased IVs were identified by the MR-PRESSO test. The Leave-One-Out analysis demonstrated the reliability of the IVW results in the absence of heterogeneity and pleiotropy (Fig 7C and 7D).

**Table 3:** MR sensitivity analysis for glaucoma’s impact on depression and anxiety.

Exposures	heterogeneity							Pleiotropy effects	
	Cochran’s Q test	MR estimates, per log-odds ratio increment after MR-PRESSO correction						MR-Egger intercept analysisi	
		Inverse variance weighted		Weighted median		MR-Egger			
	P-value	OR(95% CI)	P-value	OR(95% CI)	P-value	OR(95% CI)	P-value	Standard Error	P-value
Depression	0.085	1.000(0.998–1.002)	0.466	1.000(0.997–1.003)	0.673	1.002(0.995–1.009)	0.435	<0.001	0.560
Anxiety	0.306	1.000(0.994–1.006)	0.783	0.995(0.986–1.003)	0.245	0.985(0.968–1.003)	0.119	<0.001	0.082

## Discussion

This study provides the unidirectional perspectives on the association of glaucoma with depression and anxiety using combined meta-analysis and MR approaches. Our meta-analysis of 23 studies indicated a depression combined prevalence of 23.39% in glaucoma patients. Additionally, 21 of these studies documented an anxiety combined prevalence of 15.80% in glaucoma patients. In the cross-sectional studies, depression’s combined prevalence was 19.42% in 18 studies, and anxiety’s was 19.07% in 16 studies. In five case-control studies, glaucoma patients exhibited a 6.17 times higher risk of depression and a 4.45 times greater risk of anxiety than controls. Furthermore, two-sample MR analysis using GWAS data revealed no significant genetic causal effects of glaucoma on depression and anxiety. Sensitivity analyses demonstrated the absence of heterogeneity in the MR analyses results, thus reinforcing the reliability of our findings.

Although previous research offers insights into the association between glaucoma and depression and anxiety, they present notable limitations [26–38]. Firstly, these studies typically only provide correlational evidence, failing to explore causality, underlying mechanisms or mediating factors. Secondly, variation in study design, sample size, diagnostic criteria and assessment tools (e.g., HADS, EHR, BAI) introduce confounding variables, leading to inconsistencies in study outcomes. Additionally, research typically focuses on specific populations, potentially introducing selection bias. Lastly, the studies frequently fail to establish the temporal sequence of whether glaucoma precedes depression and anxiety or vice versa, further impacting the accuracy of the findings.

Given the significant roles of various factors such as genetics, environment, and non-genetic biological factors in the development of glaucoma, depression, and anxiety, our study presents unique advantages. In this research, the meta-analysis provides evidence of associations, while MR analysis concentrates on the role of genetic factors within these associations. The combined application of these methods aids in exploring potential associated factors based on established disease associations. This multidimensional approach is not only methodologically innovative but also enhances the depth of existing research, providing a more comprehensive perspective.

To ensure high-quality meta-analysis, stringent screening measures and comprehensive quality assessments have been implemented for the included studies. Before inclusion, each study was meticulously evaluated against our predefined stringent criteria. We used the NOS, a widely recognized tool, to precisely assess study quality. This scale helped identify methodologically sound studies and exclude those with poor design or reporting that might compromise accuracy. These steps ensured all studies met quality standards, providing a solid foundation for our conclusions. In the analysis process, despite heterogeneity, we employed sensitivity analyses to assess inter-study variability and potential publication bias. Moreover, we used a random effects model to accommodate study heterogeneity, minimizing impacts on outcomes and reducing error and bias risks. These measures ensured our meta-analysis results’ accuracy and credibility. The reliability of the MR analysis results was ensured through comprehensive complementary and sensitivity analyses.

It is essential to adopt a comprehensive and cautious perspective when considering the difference in results between two methodologies. It is also important to recognize that this difference do not reflect the reliability of either method or the quality of the included studies but highlight the multidimensionality and complexity of our understanding of disease mechanisms, due to the distinct objectives and emphases of each method. Moreover, the differences partially reflect the inherent limitations of the studies included in the meta-analysis. This limitation is due to the field’s reliance on observational studies, which inherently restricts causal inference. Consequently, although meta-analysis increases the stability and reliability of results by enlarging the sample size, it is limited to identifying statistical associations between glaucoma, depression, and anxiety. To further explore potential causal relationships and provide preliminary evidence, we utilized MR analysis. By employing genetic variants as IVs, MR analysis facilitates the inference of potential causal effects of glaucoma on depression and anxiety. However, it is also essential to recognize the limitations of MR analysis. MR analysis bases causal inference entirely on genetics and depends significantly on the robust assumptions of instrumental variables, thereby only partially alleviate the causal inference limitations of meta-analysis.

Despite employing a combined approach, our research design still has limitations. Firstly, integrating these two approaches increases the complexity of the study, especially in interpreting results, which necessitates an understanding within two distinct methodological frameworks. Secondly, Meta-analysis focuses on associations across studies, whereas MR analysis uses large GWAS databases to examine relationships between genetic variations and disease risks. Differences in data sources and research focus between the methods can lead to heterogeneity. Furthermore, genetic factors represent only a part of the mechanisms underlying disease development and do not encompass environmental or non-genetic biological pathways. Therefore, even with the combined application of both methodologies, there remain limitations in fully elucidating the complete mechanisms of the disease.

This study’s meta-analysis underscores the estimated prevalence of depression and anxiety among glaucoma patients, while MR analysis prompts us to consider the proportion of genetic factors within association. These findings are indicative rather than conclusive, suggesting that a variety of factors, including environmental elements and non-genetic biological pathways, may play a significant role relative to genetic factors, although we cannot definitively rule out the influence of genetics, further research is required to fully understand these relationships. This actually provides us with an opportunity for a more comprehensive understanding of the interactions between diseases, recognizing that explanations of these associations should not be narrowly emphasize a single factor. A more cautious approach is needed, one that takes into account the combined effects of multiple factors. This multidimensional perspective is crucial for exploring disease mechanisms and their implications.

Specifically, environmental factors may include aspects such as decreased life quality due to glaucoma, illness concerns, coping strategies and social isolation, all impacting patients’ mental health. Moreover, a patient’s socioeconomic status and social support networks also contribute. Non-genetic biological factors such as hormone levels and neurochemical changes should also be considered as potential participants. Moreover, although genetic factors were not significant in MR analysis, the role they play, along with their interactions with environmental and non-genetic biological factors, and other related factors, may still affect mental health in currently unexplored ways.

Furthermore, this finding has certain clinical implications for the comprehensive management of glaucoma, particularly in terms of regarding medication adherence and disease progression. Firstly, the complex and prolonged nature of glaucoma treatment requires patients to adhere strictly to their medication regimens to control intraocular pressure and slow vision loss, thereby maintaining disease stability. However, this challenge is magnified for glaucoma patients with compromised mental health. Deterioration in mental health, especially due to depression and anxiety, is widely reported to negatively impact medication adherence in patients [39,40]. Patients with glaucoma suffering from depression or anxiety may struggle with treatment adherence due to psychological burdens, the complexity of treatment regimens, or a pessimistic outlook on their prognosis. In this context, providing targeted psychological support and education to improve the mental health of glaucoma patients could be a key intervention to enhance medication adherence. Secondly, depression and anxiety not only alter patient behavior but may also affect glaucoma’s progression through physiological pathways. For instance, prolonged depression or anxiety may increase sympathetic nervous system activity, subsequently affecting intraocular pressure regulation, a crucial factor in glaucoma progression [4,41]. Psychological stress might also worsen glaucoma outcomes by impairing sleep quality and reducing physical activity. Therefore, regularly assessing patients’ psychological states and integrating these assessments into glaucoma management plans can help timely identify and address psychological factors that may exacerbate disease progression.

Due to limited detail in the data sources, we faced challenges in conducting detailed subgroup analyses (e.g., types of glaucoma, age, gender, geographic origins). This underscores the need for more detailed and systematic data collection and reporting methods. Although our research is preliminary, this multidimensional approach provides a new perspective for understanding the complex relationships between glaucoma and these mental health issues, emphasizing the importance of considering psychological health in the comprehensive management of glaucoma patients. Future studies should use prospective cohort designs to explore the relationships between glaucoma and mental health, considering the intricate interplay of numerous factors including environmental, genetic, and non-genetic biological factors. Research should also assess how improvements in these factors can enhance patients’ mental health. Simultaneously, investigating the effectiveness of psychological and behavioral interventions (e.g., counseling, cognitive-behavioral therapy, enhanced social support) in managing glaucoma could boost medication adherence and control disease progression, providing more effective comprehensive management strategies.

## Conclusion

This study explores the association of glaucoma with depression and anxiety from unidirectional perspectives. It reveals that the prevalence of depression and anxiety is higher in glaucoma patients than in the general population, yet it does not confirm a genetic link. It suggests that environmental factors and non-genetic biological pathways, among others, may play significant roles in their association, but it does not overlook the role of genetic factors. The study underscores the necessity of adopting a comprehensive and cautious approach in exploring the effects of glaucoma on depression and anxiety, involving a consideration of the complex interplay of multiple factors. Although preliminary, our findings highlight the crucial role of mental health considerations in the comprehensive management of glaucoma, especially regarding medication adherence and disease progression. Future research should delve deeper into identifying and quantifying the factors affecting the psychological health of glaucoma patients. It should also evaluate the potential benefits of enhancing these factors on overall mental health and their effects across various demographics. This approach will help develop more effective comprehensive management strategies for glaucoma patients, aiming to improve their quality of life and treatment outcomes.

## Supporting information

S1 Fig Flowchart of the selection for inclusion of studies in the meta-analysis. (TIF)

S1 Table Study quality assessment criteria table. (DOCX)

S2 Table Summary of identified studies and screening results. (DOCX)

S3 Table Quality assessment results table. (DOCX)

S4 Table PRISMA NMA checklist. (DOCX)
