# Supplementary figures and images for: From meta-analysis to Mendelian randomization: Unidirectional perspectives on the association of glaucoma with depression and anxiety
Source: PLoS One. 2024 Nov 19;19(11):e0310985. doi: 10.1371/journal.pone.0310985 (PMC11575789; doi:10.1371/journal.pone.0310985)

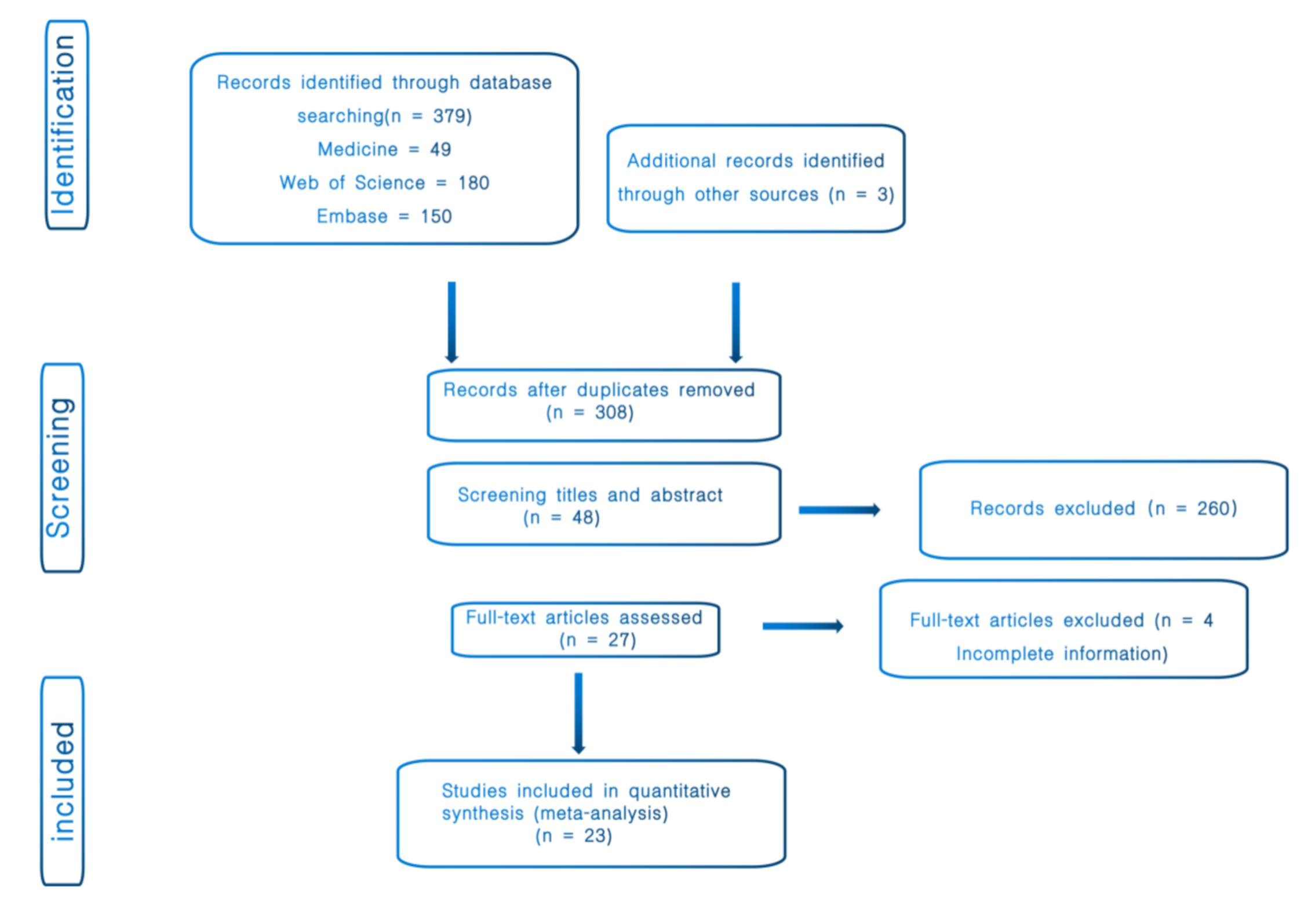

Supplement: S1 Fig — (TIF) [file pone.0310985.s001.tif]
