# Supplementary material for: From meta-analysis to Mendelian randomization: Unidirectional perspectives on the association of glaucoma with depression and anxiety
Source: PLoS One. 2024 Nov 19;19(11):e0310985. doi: 10.1371/journal.pone.0310985 (PMC11575789; doi:10.1371/journal.pone.0310985)
Supplement: S1 Table — (DOCX) [file pone.0310985.s002.docx]

S1 Table. Study Quality Assessment Criteria Table

| **Scoring Area** | **Scoring Criteria** | **Score Range** | **Remarks** |
| --- | --- | --- | --- |
| Selection | Representative Sample | 0-1 | Whether the sample represents the population (e.g., random sampling) |
|  | Adequate Sample Size | 0-1 | Whether the sample size is sufficient to ensure the reliability of results |
| Study  Design | Accuracy of Exposure/Risk Factors | 0-1 | Whether exposure/risk factors are measured accurately and consistently |
|  | Data Quality Control | 0-1 | Adequacy of data collection, quality control, validation steps, and screening |
|  | Definition of Cases and Controls | 0-1 | Whether cases and controls are defined clearly and appropriately |
|  | Selection of Control Group | 0-1 | Whether the control group is appropriately selected from the same population |
| Outcome  Assessment | Accuracy of Outcome | 0-1 | Whether the outcome is measured accurately and consistently |
|  | Timing of Outcome Assessment | 0-1 | Whether the outcome is assessed at an appropriate time point |
| Confounding  Factor Assessment | Control of Confounding Factors | 0-1 | Whether important confounding factors are considered and controlled |
|  | Stratification or Multivariate Analysis | 0-1 | Whether appropriate statistical methods are used to control confounding factors |
| Data  Analysis | Appropriateness of Statistical Methods | 0-1 | Whether suitable statistical methods for the study design are used |
|  | Reporting of Results | 0-1 | Whether results are reported completely, transparently, and reasonably |
| Study  Results | Consistency of Results | 0-1 | Whether the study results are consistent and supported by sufficient evidence |
|  | Openness and Reproducibility | 0-1 | Whether the study provides enough information to allow its results to be verified or replicated |
