# Supplementary material for: From meta-analysis to Mendelian randomization: Unidirectional perspectives on the association of glaucoma with depression and anxiety
Source: PLoS One. 2024 Nov 19;19(11):e0310985. doi: 10.1371/journal.pone.0310985 (PMC11575789; doi:10.1371/journal.pone.0310985)
Supplement: S2 Table — (DOCX) [file pone.0310985.s003.docx]

S2 Table. Summary of Identified Studies and Screening Results

| **Study**  **ID** | **研究标题** | **Study**  **Type** | **Authors** | **Publication**  **Year** | **Journal** | **Inclusion/Exclusion** |
| --- | --- | --- | --- | --- | --- | --- |
| 1 | Prevalence of depressive and anxiety disorders in patients with glaucoma: a cross-sectional study | Cross-sectional Study | Abe RY, et al | 2021 | Arq Bras Oftalmol | Inclusion |
| 2 | Cross sectional study of depression, anxiety and quality of life in glaucoma patients at a tertiary centre in North Kerala | Case-control  Study | Ajith BS, et al | 2022 | Indian J Ophthalmol | Inclusion |
| 3 | Factors associated with depression and anxiety among glaucoma patients in a tertiary hospital South-East Nigeria | Cross-sectional Study | Onwubiko SN, et al | 2020 | Niger J Clin Pract | Inclusion |
| 4 | Assessment of depression, anxiety, and quality of life in Singaporean patients with glaucoma | Cross-sectional  Study | Lim NC, et al | 2016 | J Glaucoma | Inclusion |
| 5 | Anxiety and depression in Chinese patients with glaucoma: sociodemographic, clinical, and self-reported correlates | Cross-sectional  Study | Zhou C, et al | 2013 | J Psychosom  Res | Inclusion |
| 6 | Anxiety and depression are more prevalent in primary angle closure glaucoma than in primary open-angle glaucoma | Case-control  Study | Kong X, et al | 2015 | J Glaucoma | Inclusion |
| 7 | Prevalence of depression and anxiety among participants with glaucoma in a population-based cohort study: The Gutenberg Health Study | Cross-sectional  Study | Rezapour J, et al | 2018 | BMC Ophthalmol | Inclusion |
| 8 | Anxiety, depression, and quality of life in Turkish patients with glaucoma | Case-control Study | Tastan S, et al | 2010 | Psychol Rep | Inclusion |
| 9 | High prevalence of anxiety and depression in patients with primary open-angle glaucoma | Case-control  Study | Mabuchi F, et al | 2008 | J Glaucoma | Inclusion |
| 10 | Is glaucoma comprehension associated with psychological disturbance and vision-related quality of life for patients with glaucoma? A cross-sectional study | Cross-sectional  Study | Kong XM, et al | 2014 | BMJ Open | Inclusion |
| 11 | Depression, anxiety, and disturbed sleep in glaucoma | Cross-sectional  Study | Agorastos A, et al | 2013 | J Neuropsychiatry Clin Neurosci | Inclusion |
| 12 | The effect of anxiety and depression on progression of glaucoma | Cross-sectional  Study | Shin DY, et al | 2021 | Sci Rep | Inclusion |
| 13 | Depression and quality of life in a community-based glaucoma-screening project | Cross-sectional  Study | Thau AJ, et al | 2018 | Can J Ophthalmol | Inclusion |
| 14 | Lack of perceived social support contributes to depression and anxiety in patients with glaucoma. | Cross-sectional  Study | Hamid MF, et al | 2022 | Malays J Ophthalmol | Inclusion |
| 15 | Depression and medical treatment adherence in Mexican patients with glaucoma | Cross-sectional  Study | Gamiochipi-Arjona JE, et al | 2021 | J Glaucoma | Inclusion |
| 16 | The impact of glaucoma on the mental health of primary open-angle glaucoma patients attending a teaching hospital in South East Nigeria | Case-control  Study | Ubochi CC, et al | 2020 | J West Afr Coll Surg | Inclusion |
| 17 | Illness uncertainty, anxiety and depression in Chinese patients with glaucoma or cataract | Cross-sectional  Study | Zhang D, et al | 2018 | Sci Rep | Inclusion |
| 18 | Anxiety and depression in Chinese patients with glaucoma and its correlations with vision-related quality of life and visual function indices: a cross-sectional study | Cross-sectional  Study | Wu N, et al | 2022 | BMJ Open | Inclusion |
| 19 | Analysis of life quality of primary angle-closure glaucoma patients and its influencing factors in Baotou region, Inner Mongolia | Cross-sectional Study | Li L, et al | 2021 | Chin J Exp Ophthalmol | Inclusion |
| 20 | Impact of anxiety and depression on progression to glaucoma among glaucoma suspects | Cross-sectional  Study | Berchuck S, et al | 2021 | Br J Ophthalmol | Inclusion |
| 21 | Optical quality assessed by optical quality analysis system in Chinese primary open-angle glaucoma patients and its correlations with psychological disturbances and vision-related quality of life | Cross-sectional Study | Zhang Y, et al | 2021 | Ophthalmic Res | Inclusion |
| 22 | Association between posterior segment eye diseases, common mental disorders, and depression: cross-sectional and longitudinal analyses of Brazilian longitudinal study of adult health cohort. | Cross-sectional Study | Vidal KS, et al | 2021 | J Acad Consult Liaison Psychiatry | Inclusion |
| 23 | The relationship between concomitant mental health disorders and disease severity among glaucoma patients | Cross-sectional  Study | Berchuck S, et al | 2020 | Invest Ophthalmol Vis Sci | Inclusion |
| 24 | Prevalence of Anxiety and Depression in Patients With Primary Glaucoma in Western India | Cross-sectional  Study | Dayal A, et al | 2022 | J Glaucoma | Excluded: The study reported a combined prevalence of depression and anxiety, without providing separate data for each condition. |
| 25 | The Association Between Glaucoma, Anxiety, and Depression in a Large Population | Case-control  Study | Zhang X, et al | 2022 | Indian J Ophthalmol | Excluded: The study did not provide specific prevalence data for depression or anxiety in the glaucoma patient population. |
| 26 | Evaluating the Quality of Life of Glaucoma Patients Using the State-Trait Anxiety Inventory | Cross-sectional  Study | Otori Y, et al | 2017 | J Glaucoma | Excluded: The study did not report specific prevalence data for anxiety. |
| 27 | The association between glaucoma and risk of depression: a nationwide population-based cohort study | Cohort  Study | Chen YY, et al. | 2018 | BMC Ophthalmol | Excluded: The study did not meet the inclusion criteria for study type and did not report specific prevalence data for depression in the glaucoma patient population. |
