# Supplementary material for: From meta-analysis to Mendelian randomization: Unidirectional perspectives on the association of glaucoma with depression and anxiety
Source: PLoS One. 2024 Nov 19;19(11):e0310985. doi: 10.1371/journal.pone.0310985 (PMC11575789; doi:10.1371/journal.pone.0310985)
Supplement: S3 Table — (DOCX) [file pone.0310985.s004.docx]

S3 Table. Quality Assessment Results Table

| **Study**  **ID** | **Selection** | **Study**  **Design** | **Outcome**  **Assessment** | **Confounding** | **Data**  **Analysis** | **Study**  **Results** | **Total**  **Score** |
| --- | --- | --- | --- | --- | --- | --- | --- |
| **1** | **2** | **2** | **1** | **2** | **2** | **1** | **10** |
| 2 | 2 | 3 | 2 | 2 | 2 | 1 | 12 |
| 3 | 2 | 1 | 1 | 1 | 2 | 1 | 8 |
| 4 | 1 | 1 | 1 | 1 | 2 | 1 | 7 |
| 5 | 2 | 1 | 1 | 2 | 2 | 1 | 9 |
| 6 | 2 | 3 | 2 | 1 | 2 | 1 | 11 |
| 7 | 2 | 2 | 1 | 2 | 2 | 2 | 11 |
| 8 | 1 | 2 | 2 | 1 | 2 | 1 | 9 |
| 9 | 2 | 4 | 2 | 2 | 2 | 2 | 14 |
| 10 | 2 | 2 | 2 | 2 | 2 | 2 | 12 |
| 11 | 1 | 1 | 2 | 2 | 2 | 2 | 10 |
| 12 | 1 | 1 | 2 | 2 | 2 | 1 | 9 |
| 13 | 1 | 2 | 2 | 1 | 2 | 2 | 10 |
| 14 | 2 | 1 | 2 | 2 | 2 | 2 | 11 |
| 15 | 1 | 1 | 2 | 2 | 2 | 1 | 9 |
| 16 | 2 | 4 | 2 | 1 | 2 | 2 | 13 |
| 17 | 2 | 2 | 2 | 2 | 2 | 2 | 12 |
| 18 | 2 | 1 | 2 | 2 | 2 | 2 | 11 |
| 19 | 1 | 1 | 1 | 2 | 2 | 2 | 9 |
| 20 | 1 | 2 | 1 | 2 | 2 | 2 | 10 |
| 21 | 1 | 2 | 1 | 1 | 2 | 1 | 8 |
| 22 | 2 | 2 | 2 | 2 | 2 | 2 | 12 |
| 23 | 1 | 1 | 1 | 2 | 2 | 2 | 9 |
